# New Insights into Neuroinflammation Involved in Pathogenic Mechanism of Alzheimer’s Disease and Its Potential for Therapeutic Intervention

**DOI:** 10.3390/cells11121925

**Published:** 2022-06-14

**Authors:** Tiantian Li, Li Lu, Eloise Pember, Xinuo Li, Bocheng Zhang, Zheying Zhu

**Affiliations:** 1 School of Pharmacy, The University of Nottingham, Nottingham NG7 2RD, UK; paytl4@exmail.nottingham.ac.uk (T.L.); paxll1@exmail.nottingham.ac.uk (L.L.); payerp@exmail.nottingham.ac.uk (E.P.); paybz@exmail.nottingham.ac.uk (B.Z.); 2 School of Pharmacy, China Pharmaceutical University, Nanjing 211112, China; 1831070186@stu.cpu.edu.cn

**Keywords:** Alzheimer’s disease, neuroinflammation, NF-κB, NLRP3, TREM2, cGAS-STING

## Abstract

Alzheimer’s disease (AD) is the most common form of dementia, affecting more than 50 million people worldwide with an estimated increase to 139 million people by 2050. The exact pathogenic mechanisms of AD remain elusive, resulting in the fact that the current therapeutics solely focus on symptomatic management instead of preventative or curative strategies. The two most widely accepted pathogenic mechanisms of AD include the amyloid and tau hypotheses. However, it is evident that these hypotheses cannot fully explain neuronal degeneration shown in AD. Substantial evidence is growing for the vital role of neuroinflammation in AD pathology. The neuroinflammatory hypothesis provides a new, exciting lead in uncovering the underlying mechanisms contributing to AD. This review aims to highlight new insights into the role of neuroinflammation in the pathogenesis of AD, mainly including the involvement of nuclear factor kappa-light-chain-enhancer of activated B cells (NF-κB), nucleotide-binding oligomerization domain, leucine-rich repeat-containing protein 3 (NLRP3)/caspase-1 axis, triggering receptor expressed on myeloid cells 2 (TREM2) and cGAS-STING as key influencers in augmenting AD development. The inflammasomes related to the pathways of NF-κB, NLRP3, TREM2, and cGAS-STING as biomarkers of the neuroinflammation associated with AD, as well as an overview of novel AD treatments based on these biomarkers as potential drug targets reported in the literature or under clinical trials, are explored.

## 1. Introduction

Alzheimer’s disease (AD) is a neurodegenerative disorder, often characterized by two hallmarks: amyloid plaques and neurofibrillary tangles (NFTs). The disease currently affects more than 50 million people worldwide, with a predicted increase to 139 million people by 2050 [1]. Despite its high prevalence, the pathogenic mechanisms of the disease are yet to be fully elucidated. Treatments solely focusing on symptomatic management have been developed, whilst preventative and curative strategies are yet to be introduced [2]. To reduce the prevalence and morbidity worldwide, it is necessary to gain a greater understanding of AD pathogenesis and to uncover potential therapeutic targets that can be used as preventative or curative strategies against the disease. So far, the amyloid and tau hypotheses have been the key mechanisms thought to be involved in AD pathology. There has been extensive evidence for a critical role of amyloid-beta (Aβ) in the pathology of AD over the last decades thus amyloid hypothesis largely remains the most accepted mechanism for AD pathology, yet the hypothesis does not give a full explanation for the causative mechanisms of the disease. Amyloid plaques have been found in the brain autopsies of elderly people with no AD symptoms whilst some patients diagnosed with AD have been shown to have few plaques in their brain, indicating amyloidosis may be a hallmark of aging rather than of AD itself [3,4]. Further limitations of the amyloid hypothesis can be shown by the failure of Aβ-targeted immunotherapies. Despite a reduction in Aβ concentrations in response to these therapeutics, no cognitive improvement was observed [3].

The failure of the EMERGE and ENGAGE trials to demonstrate the efficacy of the recently FDA-approved amyloid-targeting aducanumab (Aduhelm^®^) to improve cognitive function in AD patients has been extremely controversial. Biogen prematurely ended phase III clinical trials of the monoclonal antibody targeting Aβ, claiming that results had shown a significant positive trend and that an application of the therapeutic to AD was relevant. Further analysis of results from phase III clinical trials showed no treatment effect on cognitive function [5]. However, plaque-removing antibodies such as aducanumab, gantenerumab, and donanemab which target Aβ aggregation did robustly reduce the deposition of plaques in the human brain, suggesting the utility of these antibodies is in preventing rather than treatment of symptomatic AD patients [6]. Thus, FDA-approved aducanumab may bring us to an era of preventative therapy for AD. Besides, extensive evidence has indicated that soluble Aβ of oligomeric form drives neurotoxic and pro-inflammatory responses [7]. Meanwhile, recent studies revealed that the synaptotrophic effect of Aβ to modulate synaptic transmission, suggesting that selectively targeting Aβ oligomers or blocking their toxicity might be a potential preventive and curative approach for AD.

Apart from the amyloid cascade hypothesis, studies in mice or clinical trials have indicated that tau-dependent neuronal damage is initiated by Aβ, while tau increases the amyloid cascade via a feedback loop [7,8]. The amyloid hypothesis is considered the initiating factor in AD, particularly early-onset AD, however, it is recently suggested that tau pathology might be the initiating factor in late-onset AD, and it requires further investigation [9]. Despite the ambivalence of primary factors of AD, neuronal death, synaptic dysfunction, and cognitive loss that are involved in symptomatic progress are the result of the synergy of Aβ and Tau with participants of other pathological factors such as neuroinflammation.

The development of disease-modifying treatments targeting tau pathology has received arising attention in recent years, due to the less efficiency of Aβ-targeted drugs in clinical trials and lacking correlation with the severity of cognitive dysfunction. The potential therapies that target tauopathy include regulating or inhibiting post-translational modifications of tau, tau aggregation, and stabilizing cytoskeletal [10]. Memantine, an antagonist of the N-methyl-d-aspartate receptor, could activate protein phosphatase 2A (PP2A) which dephosphorylates Tau [11]. In clinical trials, memantine slightly promoted cognitive improvement while did not slow down AD progression. Furthermore, accumulating evidence indicates the preventive effect of an inhibitor of glycogen synthase kinase 3 (GSK3-β) such as tideglusib. In preclinical studies, tideglusib was reported to reduce tau phosphorylation, amyloid plaque, and cognitive improvement, however, it was found no significant improvement in cognitive functions in clinical trials [12,13]. Doubts over the efficacy of Aβ-targeting and tau pathological therapeutic may highlight the potential benefit of seeking a new approach that complements these two pathologies to prevent and delay the progression of the disease.

Neurodegeneration is a recurring characteristic in AD patients. It is first seen in the temporal lobe, affecting patients’ ability to withhold short-term memory, before occurring in the parietal lobe, restricting the ability to form long-term memory as well [14]. Therefore, cognitive impairment in these patients is correlated with neurodegeneration, highlighting its importance in understanding the symptomatic progress of cognitive decline. Treatment for AD would ideally focus on targeting neuronal degeneration to prevent the progression of the disease. Currently, there are no clinically approved drugs that inhibit or reverse neurodegeneration, mainly due to the limited understanding of the underlying mechanisms.

A study on autosomal dominant AD indicates that pathophysiological changes begin at least two decades before the clinical onset of AD, including amyloid plaques, the tau protein level in the CSF, and brain atrophy [15]. The abundant studies of amyloid and tau pathologies in AD have motivated and accelerated the development of preventive and therapeutic strategies targeting Aβ and tau, however, this focus might blur the importance and possibility of other potential pathology—neuroinflammation which becomes a hot topic recently [16]. Neuroinflammation is characterized by the activation of the immune system in the central nervous system (CNS), leading to an increased release of inflammatory molecules. Initial studies considered neuroinflammation as a secondary response followed by amyloid cascade and neurofibrillary tangles. Howbeit, recent evidence indicates that neuroinflammation is also a causative factor in AD pathogenesis, and alteration of the immune system happens earlier before the onset of AD symptoms, it is suggested that inflammation may drive cascade pathology independently of amyloid and tau pathways and interact with them, further leading to the vicious cycle [17].

Aside from the traditional Aβ and tau hypotheses, the first link between neuroinflammation and AD pathogenesis was supported by the genetic mutation of triggering receptors expressed on myeloid cells 2 (TREM2) of microglia [18]. Microglia is a central player in neuroinflammation, which drives the immune activation in the CNS. Activated microglia and released inflammatory cytokines are detected in animals and human brains with AD. Leucine-rich repeat-containing protein 3 (NLRP3) inflammasome, an important regulator in the innate immune system, is upregulated in AD brains substantially with the appearance of amyloid and tau pathology [19]. In addition, the nuclear factor kappa-light-chain-enhancer of activated B cells (NF-κB), the transcription factor that regulates many signals, is known to be the most perturbed pathway in AD, due to its essential role in neuroinflammation that is well associated with neuron growth and synaptic plasticity [20]. Furthermore, although the cGAS-STING signaling pathway plays an important role in viral infection via binding to DNA, the cGAS-STING pathway has recently emerged as an intermediary role in the process of mitochondrial stress and impaired proteostasis that induce pathological neuroinflammation and lead to neurodegeneration [21]. Recent accumulating advances in the field have shown that neuroinflammation induces neurodegeneration, indicating that treatments that significantly reduce neuroinflammation in AD patients, or those at genetic risk of AD, could revert or prevent neurodegeneration, in turn improving patient outcomes [14]. As neurodegeneration has been shown to contribute greatly to cognitive decline in AD, targeting neuroinflammation may be a more appropriate approach to improve patient outcomes in AD patients.

Although the evidence for the association of neuroinflammation with AD is strong, the underlying mechanisms are not fully understood to date. This review aims to highlight recent advances into the role of microglia in augmenting neuroinflammation in AD, including NF-κB, NLRP3, TREM2, and cGAS-STING to gain new insights into the potential therapeutic targets that could lead to novel therapeutics and substantially improve the lives of those at risk of, or suffering from, AD.

## 2. NF-κB

### 2.1. The NF-κB Pathway

**Figure 1:**
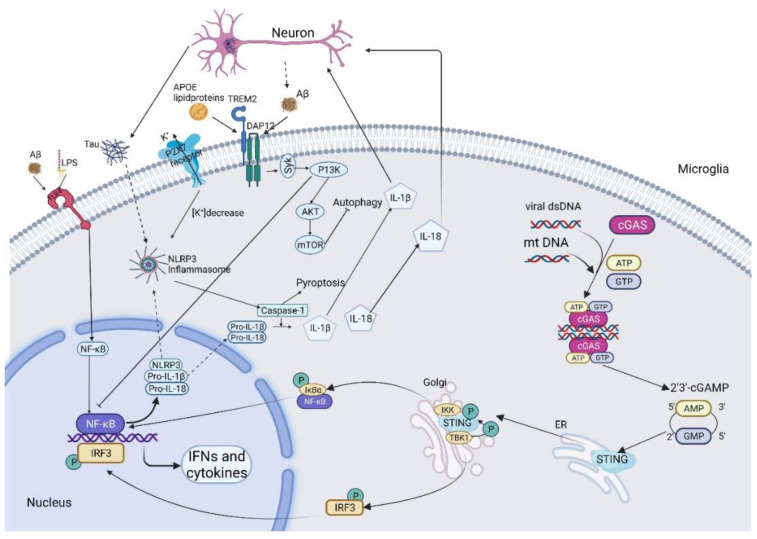
Schematic illustration indicates the role of the microglial NF-κB, NLRP3, TREM2, and cGAS-STING in Alzheimer’s disease. Aβ triggers the activation of the NF-κB pathway and NLRP3 inflammasome, leading to inflammatory cytokine release and promoting the pyroptotic death of neurons. TREM2 pairs with DAP12 through charge interactions in the transmembrane domain. Aβ and lipid proteins are the main binding ligands on the TREM2 receptor. Upon binding, DAP12 gets phosphorylated and recruits spleen tyrosine kinase (SYK), which initiates a cascade of signaling events, including phosphoinositide 3-kinase (PI3K) activation, that targets AKT and then activates mammalian target of rapamycin (mTOR), leading to inhibition of autophagy and degradation of Aβ. Foreign or mt DNA activates cGAS, which synthesizes the second messenger cGAMP from ATP and GTP. cGAMP binds to STING, leading to downstream activation of transcription factors for type I IFNs and proinflammatory cytokines that modulate neuroinflammation to produce an immune response against the pathogenic entity.

NF-κB is an important transcription factor associated with the promotion of inflammation through the upregulation of pro-inflammatory cytokines such as interleukin-1-beta (IL-1β), IL-6, IL-12, and tumor necrosis factor-alpha (TNF-α) and chemokines including IL-18 and adhesion molecules e.g., Matrix Metalloproteinase, MMP. As shown in Figure 1, activated NF-κB is often associated with amyloid plaques, highlighting its role in the neuroinflammation associated with AD [22]. Macrophages, such as microglia, have two distinct phenotypes: M1 and M2. M1 is an activated phenotype responsible for pro-inflammatory cytokine production, whilst the M2 phenotype has anti-inflammatory characteristics and is involved in producing protective mediators such as interleukin-10 (IL-10) and interleukin-13 (IL-13) [23]. In AD, microglia are seen to be chronically activated in an M1 phenotype, augmenting neuroinflammation.

Microglia can be activated via toll-like receptors (TLRs) that are presented on the cell surface. TLRs have an important role in the regulation of macrophage polarization to one of the two phenotypes [24]. In AD, the long-term activation of toll-like receptor 4 (TLR-4), by damage-associated molecular patterns (DAMPs), such as lipopolysaccharide (LPS) or Aβ, can activate the glial cell to polarize to an M1 phenotype, increasing a chronic inflammatory response [25,26,27]. This process can be seen in Figure 1. In particular, TLR signaling is activated by LPS via TLR adapters, Myeloid differentiation primary response 88 (MyD88), and TIR domain-containing adaptor inducing IFN-β (TRIF) [26]. This MyD88-dependent TLR signaling promotes interleukin-1 receptor-associated kinase (IRAK) activation, which subsequently activates self-ubiquitination, and ligation of ubiquitin to other signaling molecules [24]. These signaling molecules can activate transforming growth factor b-activated kinase 1 (TAK1), which consequently activates the inhibitor of κB (IκB) phosphorylation through the stimulation of IκB kinase (IKK) [28,29]. This phosphorylation results in the degradation of IκB, releasing free NF-κB dimers to undergo nuclear translocation, where it acts as a transcription factor to upregulate the production of M1-associated pro-inflammatory cytokines and chemokines such as IL-1β, IL-6, TNF-α, and IL-18, as well as the activation of NLRP3 inflammasome [30]. These cytokines augment the pathology of AD, creating a vicious cycle whereby the pathology of the amyloid cascade and tauopathy is accelerated. Furthermore, the TRIF-dependent TLR signaling induces Type I interferons (IFNs) [31]. This signaling pathway recruits tumor-necrosis factor receptor-associated factor 3 (TRAF3) with TRIF, followed by the activation of TANK-binding kinase 1 (TBK1) and IKKε. Therefore, TBK1 and IKKε phosphorylate Interferon regulatory factor 3 (IRF3), resulting in dimerized IRF3 which induces transcriptional activation of type I IFNs. Besides, TRIF plays a key role in NF-κB activation via adapter kinase receptor-interacting protein (RIP1), followed by RIP1 linked with Peli1 to active IKK and further triggers NF-κB pathways.

NF-κB directly upregulates the transcription of pro-inflammatory cytokines such as TNF-α and is involved in the maturation of pro-inflammatory cytokine precursors, such as pro-IL-1β and pro-IL-18 via inflammasome priming [32,33]. The primary function of these inflammasomes is to activate capsase-1, enabling the maturation of specific interleukins, such as IL-1β and IL-18, to produce a pro-inflammatory response [19]. The importance of the NLRP3 inflammasome in the promotion of neuroinflammation in neurodegenerative diseases has become well established in more recent years, however, the underlying mechanism for its regulation is still elusive [34]. There is growing evidence for the role of NF-κB in the priming of NLRP3 inflammasomes, enhancing inflammasome dysregulation, and increasing inflammatory responses to stimuli such as Aβ fibrils [32]. It is currently understood that NLRP3 inflammasome recruitment is a two-step process, NF-κB-induced upregulation of NLRP3 as an initial step, in the form of inflammasome priming, and a further stimulus causing a conformational change in NLRP3 that leads to inflammasome assembly (the second step will be discussed further later in the review) [35]. The relationship between NF-κB and NLRP3 is illustrated in Figure 1. Targeting NF-κB could allow for the downregulation of inflammasome recruitment, thus alleviating neuroinflammation in AD, which could improve cognitive function in patients.

### 2.2. Receptors for Advanced Glycation End Products (RAGE)

During neuroinflammation, receptors for advanced glycation end products (RAGE) are overexpressed on microglial cells [36,37,38]. RAGE is a multiligand receptor of the immunoglobulin superfamily and is commonly involved in binding advanced glycation end products (AGEs), but also acts as a receptor for Aβ [37,38].

The binding of Aβ to RAGE induces cytokine amplification, glutamate release, NO release, and BBB breakdown via the upregulation of NF-κB [36,37,38,39]. Cytokine amplification plays a detrimental role in neuroinflammation causing oxidative stress, amyloidogenesis, and NFTs formation. NO causes nitrosylations and further amplifies oxidative stress by combining with superoxide anions and is associated with BBB dysregulation seen in RAGE overexpression. BBB breakdown leads to an influx of peripheral monocytes and amyloid to the brain, causing further pathogenic stress [36]. The sustained release of glutamate through RAGE-mediated processes can cause chronic neuronal excitotoxicity, leading to neurodegeneration. In addition, studies have shown that upregulation of RAGE may increase RAGE-mediated peripheral Aβ transport across the BBB in a unidirectional manner, further augmenting Aβ pathogenesis, and leading to cognitive decline [40]. The blockade of RAGE has shown reduced NF-κB activity, thus RAGE-targeted therapeutics may be applicable in reducing neuroinflammation in AD [41]. Therefore, targeting RAGE may have applications in reducing both amyloidogenesis and neuroinflammation by reducing the transport of peripheral Aβ to the CNS as well as downregulating the NF-κB pathway to reduce neuroinflammation.

### 2.3. NF-κB-Targeting Drugs as Potential AD Therapeutics

NF-κB is an exciting potential target in slowing the progression of AD. It has been shown that activated NF-κB is associated with neurodegeneration in AD patients [42]. In terms of several signaling steps linked with NF-κB activation, any therapeutic approach that interferes with these steps might be a potential therapy, for example, inhibiting nuclear translocation of NF-κB, DNA interaction, phosphorylation, and degradation of IκB. Several Non-steroidal anti-inflammatory drugs (NSAIDs) that can inhibit the expression of NF-κB have been attempted in AD therapy, however, no beneficial results in AD patients were observed [43].

In other neuroinflammatory diseases, there is growing research into the use of natural products to inhibit inflammation involved in the NF-κB pathway. There is great potential for this to be extrapolated to AD to uncover a wide range of new therapeutic targets which could have preventative or curative properties. In addition, up-regulation of anti-inflammatory cytokines such as IL-10 results in increased Aβ phagocytic ability of microglia thus an improved cognitive function [44].

A wide range of studies has shown the use of phytochemicals as NF-κB inhibitors to reduce Aβ-induced neuroinflammation via this pathway [22]. Curcumin as a dietary source inhibits NF-κB, consequently reducing nitric oxide (NO) and prostaglandin E2 production. One curcumin derivative, 1,7-Bis(41-hydroxy-31-trifluoromethoxyphenyl)4-methoxycarbonylethyl-1,6-heptadiene-3,5-dione) (FMeC1), decreased Aβ deposits and improved cognitive ability in APP/PS1 transgenic mice [45]. C150, a Mannich-type curcumin derivative, suppresses NF-κB to reduce neuronal cell death and DNA mutation in brain tissue [46]. Although the mechanism of action of curcumin is still poorly understood, some studies suggest that its suppression of the NF-κB pathway is through interfering with the IκBα phosphorylation process. Despite the promising results for curcumin as a treatment for AD in animal models, results did not translate into human clinical trials [47]. This is most likely due to the lack of accurate AD brain models due to differences in underlying pathologic mechanisms in animals compared to humans, indicating that once treatments enter human clinical trials, the high complexity of the human AD brains results in the possible failure of therapeutics that were promising in pre-clinical trials. 

Similarly, another dietary source—Omega-3 polyunsaturated fatty acids—show reduced inflammatory cytokines under the inflammatory conditions induced by LPS through the NF-κB pathway. The studies suggest that omega-3 fatty acids might indirectly reduce NF-κB expression by acting as a ligand of peroxisome proliferator-activated receptor-gamma (PPAR-γ) [48].

Resveratrol is another phytochemical with current interest for its use in AD. It is an active ingredient found in the Asian herbal medicine *Polygonum cuspidatum*, commonly used for heart, liver, and gastrointestinal disorders [49]. Resveratrol can downregulate NF-κB by preventing Aβ-mediated IκB phosphorylation, thus decreasing pro-inflammatory cytokine release, including the reduction of NO and TNF-α production in microglia [50]. Evidence shows that resveratrol acts further upstream in the inflammatory cascade, preventing TLR4 oligomerization, and inhibiting NF-κB stimulation [50]. Moreover, Antonia et al. found that the anti-inflammatory effect of resveratrol also acts as an NF-κB inhibitor by upregulating IL-10 levels in LPS-stimulated microglia [51]. Unfortunately, the clinical application of resveratrol may be limited due to its low oral bioavailability (<1%) owing to extensive metabolism in the intestine and liver. Thus, formulating resveratrol as a treatment for AD may result in pharmaceutical challenges. More research into the effect of resveratrol in the human AD brain is needed before there is conclusive evidence for a clinical application.

Despite the failure of phytochemicals so far in clinical trials, these compounds still have a potential application in the development of AD therapeutics. They may serve as a useful scaffold to aid the synthesis of active analogs which could target NF-κB to reduce neuroinflammation, preventing neurodegeneration. 

Furthermore, several studies have found that other NF-κB inhibitors acting on the Tat-NEMO-binding domain, IKK-NEMO-binding domain, and the selective 5-lipoxygenase inhibitor, are involved in the therapy of brain injury, indicating their potential for AD treatment. Interestingly, suppression of the p38 MAP kinase activity prevents the loss of synapses and improves cognitive impairment related to AD. A p38α MAPK inhibitor, MW01-2-069A-SRM, has been reported to suppress proinflammatory cytokine and attenuate synaptic dysfunction and behavioral impairment in AD mice [52].

## 3. NLRP3

### 3.1. The NLRP3 Inflammasome

Inflammasomes, large cytoplasmic polyprotein complexes, can assemble by pattern recognition receptors (PRRs), including toll-like receptors (TLRs) on the cell surface which detect extracellular danger, NOD-like receptors (NLRs) that sense intracellular danger. Absent in melanoma 2 (AIM2)-like receptors (ALRs) are another group of sensor proteins. Inflammasome assembly can lead to the activation of caspase-1-mediated inflammatory responses, including cleavage and unconventional secretion of the proinflammatory cytokines IL-1β and IL-18, and initiation of an inflammatory form of cell death referred to as pyroptosis. To date, multiple inflammasomes have been found, including NLRP1, NLRP2, NLRP3, NLRP6, NLRP7, NLRC4, and AIM2 [53]. Among them, NLRP3 is the most widely studied inflammasome.

### 3.2. The NLRP3 Activation Pathway

The NLRP3 Inflammasome is a multiprotein complex, which consists of NLRP3, ASC (adaptor molecule apoptosis-associated speck-like protein containing a CARD), and caspase-1. NLRP3 is an intracellular sensor that detects a broad range of pathogen-associated molecular patterns (PAMP) and danger-associated molecule patterns (DAMP) and is implicated in the pathogenesis of several autoinflammatory diseases, including arthritis, gout, diabetes, obesity, and AD [54]. ASC acts as an adaptor protein, helping NLRP3 to recruit pro-caspase-1 via its CARD domain [55]. Active caspase-1, as an effector protein, can cleave the inactive proinflammatory cytokines pro-IL-1β and pro-IL-18 into mature IL-1β and IL-18 [56], as illustrated in Figure 1. IL-1β is a chief proinflammatory cytokine that plays a noteworthy role in the pathogenesis of neurodegenerative diseases like AD. Accumulating evidence has recognized elevated expression of IL-1β level in the activated microglia to colocalize with Aβ [57]. IL-18 also activates signaling pathways in microglia, which results in increased caspase-1 expression and pro-inflammatory cytokine production [58]. Active caspase-1 also cleaves gasdermin D (GSDMD), which allows the N-terminal domain of GSDMD to form pores in the plasma membrane, thereby triggering a lytic, pro-inflammatory form of cell death, termed pyroptosis [59], as shown in Figure 1.

The term pyroptosis has long been known to be dependent on caspase-1 since Cookson and Boise in 2001 proposed [60]. However, recent findings indicate that pyroptosis can be triggered not only by caspase-1, but also by several other inflammatory caspases, such as caspase-3, -6, -7, and -8, murine caspase-11 and its human homologs caspase-4 and caspase-5. These studies suggested that gasdermin, but not caspase-1 only, is necessary for inducing pyroptosis, because caspase-1 triggers pyroptosis by cleaving pyroptosis executors in the gasdermin protein family gasdermin D (GSDMD). Therefore, pyroptosis was defined as gasdermin-mediated programmed death in 2015 [61]. There are six genes in humans (GSDMA, GSDMB, GSDMC, GSDMD, GSDME, and DFNB59), and the gasdermin gene family in mice contains five paralogous genes but lacks GSDMB. Most gasdermin family members consist of a similar N-terminal domain and a conserved C-terminal domain, with the exception of DFNB59 [62]. The gasdermin N-terminal domain has pore-forming activity and binds to cardiolipin, phosphatidylinositol phosphates, and phosphatidylserine in the plasma membrane to induce cell death upon activation, while the C-terminal domain shows autoinhibitory activity that can suppress cell death by blocking the release of the N-terminal domain [63].

To date, studies have shown that NLRP3 inflammasomes can be activated through two different signaling pathways: canonical NLRP3 inflammasome activation and noncanonical NLRP3 inflammasome activation. It is suggested that the basal level of NLRP3 in resting cells is not sufficient to activate the inflammasome. It is widely accepted that canonical NLRP3 inflammasome activation requires two signals; signal 1 is a priming signal that can induce the transcriptional expression of NLRP3 and pro-IL [58]. In addition, emerging evidence suggests that signal 1 may also prime NLRP3 via post-translational mechanisms, such as NLRP3 deubiquitination [64]. The macrophage exposed to priming stimulus, such as TLR ligands, and cytokines like TNF-α and IL-1β, upregulates the expression of NLRP3 and pro-IL-1β by activating transcription factor NF-κB. MyD88 and TRIF, two downstream adaptor molecules of TLRs, regulate the induction of NLRP3 and pro-IL-1β transcription in response to TLR ligands during the priming phase. Signal 2 is an activating signal that can trigger NLRP3 activation, thereby promoting NLRP3 inflammasome assembly, caspase-1-mediated secretion of IL-1β and IL-18, and pyroptosis [65]. Multiple molecular and cellular events, including ion flux (such as K^+^ efflux, Cl^−^ efflux, Na^+^ influx, and Ca^2+^ mobilization), mitochondrial dysfunction, the release of reactive oxygen species (ROS) and mitochondrial DNA (mtDNA), lysosomal disruption, and trans-Golgi disassembly, can serve as a signal 2 to induce NLRP3 inflammasome activation [66]. Interestingly NLRP3 has been reported to be activated by a wide variety of unrelated PAMPs and DAMPs, but there is no evidence that NLRP3 binds directly to these effectors. 

The non-canonical inflammasome activation involves caspases 4/5 in humans and caspase-11 in mice, rather than caspase-1. These caspases sense intracellular LPS independently of TLR4 by directly binding to LPS. LPS can be recognized by Caspase-11/4/5 via direct interactions, resulting in Caspase-11/4/5 autoproteolysis and activation. Then, activated caspase-11/4/5 induces pyroptosis through two pathways. The one pathway that activated caspase-11/4/5 proteolytically cleaves murine gasdermin D (GSDMD) at Asp276 (Asp275 in human GSDMD) to induce membrane pore formation and pyroptosis. Another pathway is that the extracellular ATP released from the pannexin-1 protein channel activates the P2X7 receptor (P2X7R), an ATP-gated cation-selective channel that opens a pore to trigger K^+^ efflux, resulting in pyroptosis, IL-1β and IL-18 maturation [67].

### 3.3. The Role of NLRP3 Inflammasome in AD

The NLRP3 inflammasome expressed in microglia is the most extensively studied inflammasome, and its roles in AD are well explored. Although NLRP3 protein is also found in astrocytes, studies show that astrocytes do not express functional NLRP3, and there is no IL-1β and IL-18 secretion in astrocyte cultures in response to classical NLRP3 inflammasome stimuli [68]. This could be due to the lack of ASC and weak NLRP3 expression in these cells. It remains debated whether neurons express NLRP3. Studies in rat AD models indicate that the NLRP1 inflammasome is primarily expressed in neurons and is activated by Aβ leading to cognitive decline [69]. The NLRP2 inflammasome is present in human astrocytes. A study demonstrated that P2X7 receptor and pannexin 1 are involved in the activation of NLRP2 inflammasome [70]. Similar to the non-canonical activations of the inflammasomes, ATP released from damaged or dying cells after traumatic brain injury activates the NLRP2 inflammasome, leading to the maturation of both IL-1β and IL-18.

Aβ is the product of alternative sequential cleavage of the amyloid precursor protein (APP) by β-secretase (BACE1) followed by γ-secretase. The popular, but controversial, amyloid cascade hypothesis posits that the heavy production and deposition of Aβ into insoluble fibrils and neurotoxic plaques drive the neurodegenerative process [64]. In fact, the oligomeric forms of Aβ are potentially more neurotoxic compared to their fibrillar counterparts. Aβ oligomers and fibrils are known inducers of neuroinflammation, owing to their ability to potentiate inflammasome activation. Deposition of Aβ drives cerebral neuroinflammation by activating microglia. Indeed, a study by Halle et al. provided preliminary evidence that Aβ aggregates activated the NLRP3 inflammasome in microglia, leading to IL-1β and IL-18 production and the generation of an inflammatory milieu [71]. Persistent microglial NLRP3 inflammasome signaling triggers microglial dysfunction, thus reducing the capacity of microglia to clear Aβ and NFTs, creating a destructive cycle of Aβ and tau accumulation and microglial activation [72]. Recent studies show that severe AD is associated with increased microglial dysfunction compared to healthy control brains [73]. Moreover, NLRP3-mediated neurotoxicity was also observed in the in vivo mouse model challenged with Aβ oligomers. The study demonstrated that NLRP3- or caspase-1-deficient APP/PS1 mice were protected from neuroinflammation, amyloid plaque deposits, and AD-related pathology [74]. In contrast, NLRP3 inflammasome activation exacerbates amyloid pathology in vivo by prion-like ASC-speck cross-seeding [75].

Tau is a microtubule-associated protein expressed in neurons that undergoes a variety of post-translational modifications including hyperphosphorylation and aggregation to form intracellular NFTs, which ultimately drives neuronal toxicity and death in AD. Ising et al. reported that NLRP3 deficiency could decrease tau hyperphosphorylation and aggregation via mediating tau phosphatases and kinases. Furthermore, tau pathology could be induced by intracerebral injection of brain homogenate containing fibrillar Aβ in an NLRP3-dependent manner, indicating that NFTs are a downstream pathway of Aβ-induced microglial activation [76]. In summary, tau pathology is highly associated with the activation of the NLRP3 inflammasome in AD, as illustrated in Figure 1.

### 3.4. Therapeutic Targeting NLRP3 in the Treatment of AD

Accumulating evidence has indicated the role of NLRP3 inflammasome in AD pathogenesis. Direct suppression of the activation of the NLRP3 inflammasome, or targeting relevant components, has great prospects for the prevention and treatment of AD. To date, only 2 categories of medicines have been licensed for AD, including AChE inhibitors and memantine. Among them, donepezil is considered the most cost-effective AChE inhibitor [77]. Kim et al. revealed the therapeutic effect of donepezil in its response to LPS- and Aβ-induced neuroinflammation by suppressing AKT/MAPK, NLRP3 inflammasome, and NF-κB/STAT3 signaling in vitro and in vivo, suggesting that donepezil was a therapeutic agent for neuroinflammation-associated diseases such as AD [78].

MCC950, also named CRID3, was reported as a selective and effective inhibitor of the NLRP3 inflammasome both in vivo and in vitro [79]. Dempsey et al. observed the therapeutic effect of MCC950 in APP/PS1 mice and suggested that MCC950 could improve cognitive function and promote Aβ clearance, indicating that the inhibition of the NLRP3 inflammasome was a crucial therapeutic target for AD [80]. Additionally, in a tau transgenic mouse model, chronic intracerebral injection of MCC950 was shown to inhibit exogenous tau pathology [81]. Meanwhile, MCC950 also showed a potential effect under hyperlipidaemic conditions. Overall, MCC950 treatment resulted in a strong reduction in the IL-1β secretion in Apolipoprotein E deficient mice by inhibiting the NLRP3 inflammasome. MCC950 is therefore a potential treatment strategy to target the NLRP3 inflammasome in AD and deserves to be further evaluated in animal experiments and clinical trials. 

Except for MCC950 as a direct inhibitor of NLRP3 protein, CY-09, OLT1177, and Oridonin also exhibit potential roles of anti-neuroinflammation in NLRP3-involved diseases. CY-09, an analog of cystic fibrosis transmembrane conductance regulator (CFTR) inhibitor-172 (C172), blocks ATP binding with NLRP3 by directly binding to the NLRP3 Walker A motif, showing protective effects in mice models of gout and Types 2 diabetes. CY-09 has a promising potential as an NLRP3 inhibitor with good oral bioavailability and pharmacokinetic profile [82]. 

Dapansutrile (OLT1177) is an active β-sulfonyl nitrile small molecule that prevents activation of the NLRP3 inflammasome by directly binding and inhibiting NLRP3 ATPase activity. In vitro analysis observed that nanomolar concentrations of OLT1177 directly reduced IL-1β and IL-18 release [83]. Lonnemann et al. reported that APP/PS1 mice treated with OLT1177 showed rescue effects in various assessments, ranging from improved cognitive function to overall reduction in proinflammatory cytokines in the brain, suggesting the potential benefits of pharmaceutically blocking NLRP3 signaling in AD [84]. The pharmacokinetic and safety profile of OLT1177 has already been confirmed in a Phase I clinical trial, indicating it may be a possible therapeutic option for AD. 

Oridonin is derived from the herbal plant *Rabdosia rubescens*, it is reported to attenuate Aβ_1-42_-induced neuroinflammation by inhibiting the NF-κB pathway [85]. Furthermore, He et al. found that Oridonin inhibits activation of the NLRP3 inflammasome through binding with the NLRP3 NACHT domain to block NLRP3-NEK7 interactions, suggesting that Oridonin might be a promising treatment agent for AD-related neuroinflammation [86].

Fenamate-type NSAIDs were found to selectively inhibit the NLRP3 inflammasome [87]. In a model of Aβ-induced memory loss and a transgenic mouse model of AD, fenamate NSAIDs inhibited cognitive impairment and demonstrated both protective and therapeutic efficacies. Diclofenac, a structurally related NSAID, was similarly shown to reduce levels of IL-1β [88]. Based on the clinical availability of fenamate NSAIDs, repurposing them as NLRP3 inhibitors may provide a rapid therapeutic AD treatment strategy.

Pyrin-only proteins (POPs) and CARD-only proteins (COPs) as physiological inhibitors were found to have a role in hindering neuroinflammation/neurodegeneration [89]. Inflammasome assembly relies upon homo domain interactions between the structurally related Pyrin and caspase-recruitment (CARD) domains and adaptor proteins, such as ASC, or effector proteins, such as caspase-1. 

The study discovered three human encoded POPs, including POP1, POP2, and a potential POP3, and three COPs (CARD16, CARD17, and CARD18), initially described their ability to inhibit caspase-1 activity. POPs and COPs are relatively short proteins with approximately 90 amino acids composed essentially of only a PYD or CARD domain. The study demonstrated that the macrophage-specific expression of POP1 impairs ASC-dependent inflammasome responses by binding to ASC and thereby preventing ASC-PRR interactions, which ultimately ameliorates systemic inflammation and auto-inflammatory disease [90]. POP3 functioned as a key regulator of ALR inflammasomes in human and mouse macrophages in vivo [91]. Although POP3 does not bind to ASC, it specifically interacts with the cytosolic DNA sensors AIM2 [91]. These studies suggest that POP3 can inhibit inflammasome activation and promote type I interferon production. The third member, POP2, is unique, as it not only binds to ASC to inhibit inflammasome activation, but also inhibits NF-κB activation of the inflammasome priming stage in vitro [92]. However, its inflammasome and NF-κB-inhibiting roles have not been investigated in detail, and the function of POP2 in vivo is still elusive. 

CARDs are known for their ability to oligomerize and interact with other CARDs [93]. Accordingly, CARD16 can self-oligomerize, similar to the CARD of caspase-1, forming filament-like structures. CARD also interacts with the CARD of ASC, co-localizing in perinuclear ASC specks. Taken together, this suggests that CARD16 could regulate inflammasome assembly, caspase-1 activation, and cytokine maturation. Although CARD17 is unable to self-oligomerize and cannot interact with ASC-CARD, similar to CARD16, CARD17 is also able to bind to the CARD of caspase-1 and interferes with the self-oligomerization of the caspase-1 CARD, suggesting that CARD17 could have an inhibitory effect on caspase-1 activation. CARD 18 plays a controversial role in regulating inflammasome activation. The study indicated that CARD18 inhibits caspase-1 oligomerization and activation by CARD-CARD interaction through low homology to the CARD of caspase-1. Another report proposed the opposite effect of CARD18 that CARD18 actually promotes caspase-1 polymerization and filament formation, thereby activating caspase-1. Despite recent advances, further work would be needed to provide deeper insights into the role of COPs and POPs in controlling inappropriate inflammasome responses.

## 4. TREM2

The genome-wide associated studies (GWAS) of AD reveal several genetic risk factors in AD development, in which variants of TREM2 have been found to increase AD risk 2–4 folds, similar to the risk of patients with one copy of ApoE4 [94]. The loss of function studies after knock-out TREM2 in mice further shows the crucial role of TREM2 in AD pathology [95]. In the brain, TREM2 is primarily expressed in the microglia that regulate cerebral homeostasis by giving neurotrophic support and phagocytosis of cell debris and amyloid in neurons [96]. Most interestingly, TREM2 expression varies in different regions of the brain. In particular, it is highly expressed in the hippocampus which is partially consistent with the pathological features of AD [97]. Increased TREM2 expression has been detected in AD patients and AD mice models and has also been found in activated microglia [98,99]. Therefore, a better understanding of the functions of TREM2 in AD, and the potential therapies targeting TREM2, deserve further investigation.

### 4.1. TREM2 Structure and Variants Related to AD

TREM2 is a transmembrane protein with a V-immunoglobulin domain. The intracellular activity of TREM2 acts through binding with DAP12 (DNAX-activation protein 12) by a lysine-aspartate electrostatic interaction, as shown in Figure 1. Knockdown of TREM2 or DAP12 in microglia resulted in reduced phagocytosis of apoptotic neurons [100].

In addition, the function of TREM2-DAP12 needs the participation of ligands that are bound with the extracellular domain of TREM2. The exact ligands of TREM2 are uncertain, though LPS, lipoteichoic acids, lipids, and Aβ have been found to activate TREM2 signaling [101]. Several studies show that lipids are bound with TREM2 from the cell membrane and lipoprotein complex. Given this fact, APOE raises much attention due to its main expression in the brain. The APOE-TREM2 binding is independent of lipidated states of APOE, albeit the lipidated APOE has a higher affinity than non-lipidated APOE [102]. Other lipoproteins, such as high-density lipoprotein (HDL) and low-density lipoproteins (LDL), have also been reported as ligands of TREM2.

Apart from its role as a membrane receptor, TREM2 contains a long ectodomain that is processed by the disintegrin and metalloprotease ADAM10 or ADAM17 to release a soluble TREM2 (sTREM2) which can be detected in plasma and cerebrospinal fluid (CSF) [103]. The initiation and activity of TREM2-related signaling and function require the form of full-length TREM2, although recent data show that sTREM2 may also have its own biological capability of binding ligands and activating TREM2 signaling [104]. Furthermore, several clinical trials found an increased level of sTREM2 in CSF of AD patients, suggesting the potential role of sTREM2 as a biomarker, which requires further study [105].

Previous studies identified several variants of TREM2, including R47H, R62H, T66M, Y38C, T96K, and D87N [106]. Among them, rs75932628-T was considered to have the strongest association with AD, showing substitution of histidine by arginine at position 47 (R47H) in the gene encoding TREM2 in chromosome 6p21.1. The R47H mutation damages the ligand-binding domain of TREM2 which increases the risk of AD by 2–4.5 folds [107]. TREM2 signaling was inhibited in variant R47H, which causes similar transcriptional dysregulation to knockout TREM2 in iPSC-derived macrophages [108]. 

### 4.2. TREM2 Functions in AD Pathology

Currently, two signaling streams of TREM2 are considered to regulate the reactive phenotype, including phagocytosis and anti-inflammatory effects, in which TREM2 shows the ability to increase the phagocytosis of damaged neurons, cellular debris, and Aβ. The function of Aβ clearance has been associated with TREM2 based on evidence that TREM2 knock-out decreased the uptake and degradation of Aβ [109]. In vivo studies of the amyloid mouse model reveal the crucial function of TREM2 in the clustering microglia around plaques, plaque compaction, and microglial proliferation.

The association between TREM2 and tau pathology was investigated by studies in which TREM2 knock-out dramatically enhanced Aβ-induced tau seeding and spreading around plaques in the amyloid mouse model [110]. Conversely, tauopathy studies in PS19 mouse models suggest that TREM2 deficiency attenuates neuroinflammation, while TREM2 itself protects against tau-mediated neurodegeneration. It was shown that TREM2R47H Variant PS19 mice consistently had significantly attenuated brain atrophy, synapse loss, and microglial reactivity versus the common variant TREM2 mice [111]. In another study, Bemiller et al. built a cross transgenic TREM2-deficient human tau (hTau) mouse model. They showed that a decrease in microgliosis and complete deletion of TREM2 exacerbated tau pathology [112]. It is noted that the varied functions of TREM2 in microglia under various pathological conditions of AD need further investigation.

Moreover, the expression of TREM2 is affected by inflammatory conditions, for example, anti-inflammatory molecules enhance its expression while pro-inflammatory cytokines like LPS, TNFα, and IL1β reduce its expression [113]. In a primary microglia study, LPS largely suppressed TREM2 expression as early as 3 h after LPS treatment, indicating that the LPS/IFNγ activation reduced TREM2 via TLR4-induced pro-inflammatory pathway [114]. TREM2 has an anti-inflammatory function. It inhibits macrophage response to ligation of TLR, and it negatively regulates TLR-mediated maturation of dendritic cells, type I interferon responses, and the induction of antigen-specific T-cell proliferation. Furthermore, TREM2 stimulation of dendritic cells induces partial activation without any production of pro-inflammatory cytokines. The anti-inflammatory function of TREM2 is associated with the IL-4 stimulation pathway by promoting levels of the transcription factor STAT6, involving genes such as Arg1, Ap1b1, and Dusp4 [115]. Recent research revealed that the PI3K/AKT/FoxO3a signaling pathway is involved in TREM2-mediated inflammatory response to ameliorate neuroinflammation and cognitive impairment [116]. The microglial study in LPS-induced mice concluded that the neuroinflammatory inhibition of TREM2 is regulated by down-regulating PI3K/AKT and NF-κB signaling [117]. More studies show that TREM2 can inhibit the release of inflammatory factors from LPS-stimulated microglia by inhibiting NF-κB signaling pathway activity [118], and autophagy by PI3K/AKT pathway [119]. The process is illustrated in Figure 1.

Taken together, the expression of TREM2 differs during different stages of neuroinflammation. The increased levels of TREM2 may result from the recruitment of microglia for clearance of Aβ and anti-inflammatory reactions.

### 4.3. Targeting TREM2 as a Novel Strategy for AD

Given the fact that stimulating the activity of TREM2, or increasing the expression of TREM2, protects neurons under the AD pathological state, novel strategies targeting TREM2 signaling have been developed in preclinical and clinical studies [120]. Stereotaxic injection of recombinant sTREM2 in the brain of 5xFAD mice demonstrate the reduction of Aβ and improvement of cognitive function [121]. Interestingly, sTREM2 enhances microglial proliferation uptake and degradation of Aβ, while depletion of microglia inhibits the neuroprotective effects of sTREM2 [122]. It is suggested that the therapeutic effect of TREM2 may be produced through regulating microglia. However, sTREM2 has also been found to be associated with tau pathology evidenced by the increased level of sTREM2 in the CSF related to a higher level of phosphor-tau181P in AD patients [105,123]. These two contradictory views on sTREM2 might result from the difference between a human brain and a rodent’s brain. While the roles of TREM2 played in AD pathology have been elucidated, the impact and mechanism of sTREM2 on amyloid and tau pathologies remain to be further determined.

Another strategy to enhance TREM2 expression or activity is by reducing proteolytic shedding of the TREM2 extracellular domain by A Disintegrin and Metalloproteinase (Adam) 10/17 (α-secretase), due to the phagocytic capacity of microglia increased by inhibiting Adam proteases [124]. However, inhibition of α-secretase might affect the release of neurotrophic and neuroprotective soluble AβPPα and enhance the production of senile plaques, which needs to be considered carefully. For this reason, researchers are seeking drugs or antibodies that can selectively inhibit TREM2 shedding without affecting the cleavage of sAPP and the shedding of other ADAM 10/17 substrates. By selectively preventing α-secretase shedding of TREM2, several monoclonal antibodies have been screened, in which 4D9 was selected due to its ability to stabilize TREM2 on the cell surface and prevent proteolytic shedding. It has been demonstrated in vivo that 4D9 reduced amyloidogenesis and enhanced TREM2 expression [125].

AL002, a humanized monoclonal IgG1 antibody, is being developed by Alector and AbbVie which could activate TREM2 signaling and increase phosphorylation of Syk through binding to microglial receptor TREM2. AL002a, a variant of AL002 that targets the TREM2 signaling pathway, increased CD11b-positive microglia in the cortex and hippocampus of APP/PS1 mice, enhanced microglial clustering at amyloid plaques, and reduced amyloid deposition in 5×FAD mice [126]. AL002c, another variant of AL002 further modified for clinical use, was tested in human TREM2 gene-carrying 5×FAD mice and indicated microglial activation as well as reduction of inflammatory signaling and neurotoxicity. AL002c has shown to be safe and well-tolerated in phase I clinical trials; the phase 2 trials started in 2020 and will finish in 2023 [127,128].

Yuhai et al. found that NF-κB-sensitive miRNA-34a was upregulated in sporadic AD patients, consistently with downregulated TREM2 expression [129], suggesting the potential therapeutic role of miRNA-34a for regulating innate immune and phagocytic responses in neuroinflammation. Heat shock protein 60 (HSP60), a mitochondrial chaperone, is suggested as a TREM2 agonist, leading to the activation of TREM2 signaling and enhancement of microglial phagocytosis [130]. However, the underlining mechanism of the effect of HSP60 in AD therapy is unclear. Apart from the fact that increasing TREM2 expression may represent potential therapies for AD, inhibiting CD33 may also show a protective impact due to TREM2 acting downstream of CD33 in the modulation of microglial pathology in AD [131]. In an in vivo study using 5×FAD mice, CD33 knockout reduced Aβ pathology and mitigated memory loss, conversely both of which were abrogated by knockout TREM2. The invention of ligand mimetic peptides could also exhibit a novel therapeutic approach for AD. COG1410, an APOE-mimetic peptide, improved neurological function, and attenuated neuroinflammation and neuronal apoptosis by regulating the PI3K/Akt pathway in a mouse model of intracerebral hemorrhage [117].

## 5. cGAS-STING Pathway

### 5.1. The cGAMP-STING-IRF3 Signaling Pathway

The cGAS-STING pathway has emerged as a vital mechanism for coupling the sensing of pathogenic DNA to the stimulation of the innate immune defense program. The cyclic guanosine monophosphate (GMP)-adenosine monophosphate (AMP) synthase (cGAS) is an innate immune system receptor that detects pathogenic DNA and alerts the immune system to infectious agents. The stimulator of interferon genes (STING) is an innate immune adaptor protein that acts in concert with cGAS to mount an interferon-based response to protect the host. Within this pathway, the binding of cGAS to cytosolic double-stranded DNA (dsDNA) activates its catalytic activity and converts ATP and GTP to a cyclic dinucleotide 2′3′-cyclic GMP-AMP (cGAMP) [132]. The cGAMP is a second messenger that binds to and activates the stimulator of interferon genes (STING) located in the endoplasmic reticulum (ER). The bound cGAMP changes STING conformation. Thus, the STING oligomer is translocated to perinuclear regions and binds TANK-binding kinase 1 (TBK1), where it recruits and phosphorylates the transcription factor—interferon regulatory factor 3 (IRF3). IRF3 is a master regulator of Type I interferons. It is further translocated to the nucleus and activates the transcription of genes encoding interferons and other cytokines, resulting in the production of Type-I IFNs and some inflammatory cytokines. Activation of STING also leads to NF-κB activation, resulting in subsequent signaling transductions, as illustrated in Figure 1.

### 5.2. Effects of cGAS-STING Pathway on Neuroinflammation

The cGAS/STING/IFNs axis has emerged as a critical signaling pathway of the innate immune system in the CNS. The abundance of cGAS/STING has been identified in the context of neuroinflammation-related disorders. Interferons that the cytokines produced by the STING pathway are classified as Type I (IFN-I), Type II (IFN-γ), and Type III (IFN-λ). Early studies revealed that the brain is more vulnerable to the effects of Interferons though the low levels of interferon-stimulated gene (ISG) mRNAs were expressed in the CNS [133]. Among various Interferons, the involvement of Type I interferons (IFN-I) in neuroinflammation has long been speculated. In AD and aging, IFN-γ signaling is suppressed while IFN-I activity is elevated, thus suppression of IFN-I signaling is beneficial. IFN-I signals via the heterodimeric receptor (IFNAR) and Janus kinase (JAK)/signal transducer and activator of transcription (STAT) signaling cascade [134]. IFN-I binds to their respective receptors on microglial cells, astrocytes, and neurons. Microglial cells are activated and respond to interferons to communicate with neurons. Activated microglia secrete cytokines and chemokines, several of which, such as IL-1β and TNF-α, elicit neurotoxicity. Subsequently, cytokines TNF-α and IL-1β released from activated microglia cause an increase in the production of nitric oxide (NO) and superoxide, peroxynitrite (ONOO-) via some enzymes in astrocytes. Over-activation of glial cells elicits overproduction of cytokines, which mediates neurodegeneration by activation of p53, in addition to NF-κB, leading to neuronal loss and neurodegeneration [135].

Chronic over-activation of cGAS-STING promotes inflammation that accelerates a variety of aging-related diseases, but inhibition of this pathway could increase vulnerability to infection. In healthy organisms, the cGAS-STING pathway is regulated by autophagy as a cell-autonomous defense mechanism [136]. Autophagy regulates cGAS activity by its autophagy protein beclin-1. Its interaction with beclin-1 can promote the autophagic degradation of both cytosolic DNA and cGAS itself and inhibit the production of cGAMP, the second messenger that drives IFN production [137]. However, type 1 IFN can disrupt the autophagic degradation of cGAS by inducing the E3 ligase TRIM14 to stabilize cGAS [138]. STING is also regulated in an autophagy-dependent manner. Activation of STING can trigger canonical autophagy response relying on the ULK complex and TBK1, and a non-canonical autophagy response requiring only selective autophagy machinery components, including the PI3P effector WIPI2 and the ATG5-12-16L1 complex [139]. STING itself also becomes a target for degradation, which prevents over-activation. Therefore, cGAS-STING signaling is sensitive to the cellular environment, thus this pathway can become chronically activated in the context of elevated inflammation and disrupted autophagy, as is common in many neurodegenerative diseases.

AIM2 inflammasomes play an important role in the negative regulation of the cGAS-STING pathway due to their ability to sense cytosolic DNA [140]. AIM2 has one HIN domain and one PYD domain. Under homeostasis, AIM2 exists in an autoinhibitory stage due to an interaction between HIN and PYD domains that block the availability of PYD for ASC PYD. AIM2 can be activated by mtDNA, nuclear DNA released in the cytosol due to nuclear death, and self-DNA secreted by exosomes. Upon binding to the cytosolic dsDNA with 80–300 bp by the HIN domain, AIM2 recruits ASC protein via the PYD domain, while ASC protein recruits pro-caspase-1 (pro-CASP1) via the CARD domain. The pro-caspase-1 filament polymerization activates caspase 1 (CASP1) through auto-proteolysis to produce pro-inflammatory cytokines (IL-1β and IL-18) and induce pyroptosis. Pyroptosis in AIM2 inflammasomes is an important process to prevent cGAS-STING signaling and type 1 IFN production. Activated CASP1 also cleaves the linker region of the GSDMD and releases GSDMD domains (GSDMD-N and GSDMD-C), leading to pore formation and K^+^ efflux from this pore. The K^+^ efflux from the GSDMD pore inhibits cGAS activity and STING-TBK1 interaction required for IRF3-dependent type 1 IFN release. Therefore, AIM2-induced GSDMD acts as a negative regulator of cGAS-STING-mediated type 1 IFN production. Interestingly, unlike the positive effect of knock out of NLRP3, recent evidence shows that AIM2 deletion does not have a beneficial effect on the spatial memory or cytokine expression of 5xFAD mice though it mitigates Aβ deposition and microglial activation [141]. The reason for the reduction of Aβ deposition is suggested by studies that activation of the T1 IFN response by AIM2 deletion in microglia increased TREM2 mRNA levels [142], which encodes for the TREM2 receptor that regulates microglial functions (including phagocytosis of Aβ deposits), it is conceivable that the AIM2 gene deficiency reduced the Aβ deposits in the AIM2^−/−^ (B6.Sv129) mice [143].

### 5.3. The Role of cGAS-STING Signaling in AD

It has been known that mitochondrial damage is a key factor in the activation of cGAS-STING signaling when cGas acts as a DNA sensor. The activation of the cGAS-STING signaling pathway is not only by foreign DNA, but also via inner DNA such as mitochondrial DNA (mtDNA). Aberrant mitophagy can lead to the accumulation of DNA. Release of mtDNA into cytosol triggers cGAS, activation of STING, and ultimately induction of the type 1 interferon (IFN) and interferon responsive genes [144]. Therefore, the cGAS-STING pathway is considered an intermediary in the process of mitochondrial stress-induced neuroinflammation, leading to neurodegeneration. Cox et al. demonstrated in vivo that both astrocyte and microglial cells express the enzyme cGAS at mRNA level [145] and found that microglia upregulate cGAS in response to IFN-β treatment and induce more production of type 1 IFN and pro-inflammatory cytokines. Xu et al. discovered that the STING stimulator, cGAMP, significantly reduced pro-inflammatory cytokines (IL-1β, TNFα) and converted the M1 phenotype towards the M2 phenotype of microglia by increasing the expression of TREM2 in 5-month-old APP/PS1 AD mice [146]. While NLRP3 inflammasome acts as a well-studied pathway of the innate immune responses, Wang et al. revealed a distinct mechanism by which STING regulates the NLRP3 inflammasome activation, IL-1β secretion, and inflammatory responses in human cell lines, mice primary cells, and mice [147]. Furthermore, Jin et al. identified the interaction of polyglutamine binding protein 1 (PQBP1) with tau 3R/4R proteins, triggering an innate immune response via activating the cGAS-STING pathway [148]. PQBP1 in microglia could be a promising novel target for therapeutics against AD and tauopathy. Although the over-activation of cGAS-STING signaling promotes neuroinflammation, resulting in AD pathologies, the effect of cGAS-STING signaling in AD progression is complex, which depends on the different roles of neuroinflammation in different stages of the disease.

### 5.4. Therapeutic Targeting of cGAS-STING Pathway in Treatment of AD

There are numerous preclinical studies of cGAS-STING activators designed to treat cancer, and some designed to treat autoimmune diseases [149]. Accumulating evidence has indicated the cGAS-STING pathway in driving neuroinflammation of neurodegenerative diseases, thus inhibition of this pathway represents a viable therapeutic in the treatment of neurodegenerative diseases. However, while cGAS-STING inhibitors may protect against inflammation-driven diseases of aging, they may promote acute infection and cancer due to prolonged suppression of the neuroinflammatory response. Given the multifaceted role of STING in each disease context, it is important to know the optimal therapeutic window for STING activation and inhibition specifically, to develop a safe and effective STING modulator. Although there are currently no cGAS-STING inhibitors under preclinical research for AD, in the findings described above there are some potential therapeutic targets including cGAMP [146] and PQBP1 [148] to be further validated as novel drug targets in AD. Both targets show neuroprotection in vivo, and their inhibitors could be a potential treatment strategy for AD in the future. In a recent study using the APP/PS1 mutant transgenic mice model, Hou et al. firstly found that NAD+ levels reduced, and markers of inflammation increased. Following treatment with the NAD+ precursor nicotinamide riboside (NR), the reduced level was reversed to the normal. NR treatment can further normalize cGAS-STING upregulation in mice models, and so NAD+ supplementation could be considered a drug candidate for treating AD via cGAS-STING [150].

## 6. Conclusions

This review has given an overview of new insights into the role of neuroinflammation in AD. The upregulation of the pathways of NF-κB, NLRP3, and cGAS-STING, along with the downregulation of TREM2, has been shown to have a significant impact on the augmentation of AD pathology. The overlapping pathways between NF-κB, NLRP3, TREM2, and cGAS-STING indicate that pharmacological intervention in any one of these factors could lead to significant amelioration of neuroinflammation, thus cognitive improvement in AD patients.

Substantial evidence has shown that there is, undoubtedly, a major role of neuroinflammation in the progression of AD. Currently, in vitro and in vivo studies have provided evidence for the benefit of reducing neuroinflammation in AD and other neuroinflammatory disorders; however, there has not been any success in progressing any of these treatments to clinics, albeit a few potential products under clinical trials. Failure of successful anti-inflammatory treatments for AD may be due to a lack of comprehensive understanding of the mechanisms of neuroinflammation. Full elucidation of the neuroinflammatory processes underlying the diseased state could unlock novel targets for more efficacious treatment improving AD-associated morbidity.

There is a predominantly prevalent role of amyloidosis and tauopathy in AD pathology, while neuroinflammation may provide a novel mechanism for disease progression, at least a supplement for the traditional concept of AD pathology. Therefore, a more synoptic approach to AD pathogenesis is necessary to understand the true underlying causes of the disease. The review based on evidence summarizes a cyclical process between amyloidosis, tauopathy, and neuroinflammation as Figure 1 illustrates. It is commonly thought that the dysregulation of amyloid processing, leading to increased Aβ concentration, is the initiating factor in AD pathology. Aβ then seeds further pathology by causing tauopathies and subsequent NFTs, as well as increasing neuroinflammation. This neuroinflammatory response can further augment amyloidosis and tauopathies leading to cognitive decline. However, cGAS-STING suggests a novel route to cause neuroinflammation through infection-activated microglia, triggering the NF-κB pathway and other inflammasomes, as the primary driver of AD. Therefore, combinatory treatment strategies that would target neuroinflammation, Aβ, and tauopathies all could be more effective interventions for AD with such complicated pathological mechanisms.

This review suggests that new insights into the neuroinflammatory response can provide drug targets adequate in reducing cognitive decline. The pharmacological inhibition of NF-κB, NLRP3, and cGAS-STING, or enhancement of TREM2 in AD patients, could lead to substantial improvement in patient outcomes.

This review is mainly focused on microglia and limited to astrocytes and neurons involved in pro-inflammatory effects, together with the difference between human and rodents’ brains that play roles in the complex AD neuropathology being not included.

## Data Availability

Not applicable.
